# Mapping the research trends of astrocytes in stroke: A bibliometric analysis

**DOI:** 10.3389/fncel.2022.949521

**Published:** 2022-09-08

**Authors:** Zhibin Ding, Nan Jiang, Ting Yang, Hongxia Han, Miaomiao Hou, Gajendra Kumar, Yige Wu, Lijuan Song, Xinyi Li, Cungen Ma, Yanbing Su

**Affiliations:** ^1^Department of Neurology, Third Hospital of Shanxi Medical University, Shanxi Bethune Hospital, Shanxi Academy of Medical Sciences, Tongji Shanxi Hospital, Taiyuan, China; ^2^The Key Research Laboratory of Benefiting Qi for Acting Blood Circulation Method to Treat Multiple Sclerosis of State Administration of Traditional Chinese Medicine, Research Center of Neurobiology, Shanxi University of Chinese Medicine, Jinzhong, China; ^3^Shanxi Cardiovascular Hospital, Shanxi Medical University, Taiyuan, China; ^4^Department of Neuroscience, City University of Hong Kong, Hong Kong, Hong Kong SAR, China; ^5^General Surgery Department, Third Hospital of Shanxi Medical University, Shanxi Bethune Hospital, Shanxi Academy of Medical Sciences, Tongji Shanxi Hospital, Taiyuan, China

**Keywords:** stroke, astrocytes, bibliometric analysis, citation analysis, scientific outputs

## Abstract

**Background:**

Stroke, including ischemic stroke and hemorrhagic stroke, possesses complex pathological mechanisms such as neuroinflammation, oxidative stress and blood-brain barrier damage. Astrocyte functions have been reported during injury, neuroprotection and cell crosstalk. It plays a key role in exacerbating stroke injury, promoting neurological repair and enhancing neuroregeneration.

**Aim:**

This holistic bibliometric analysis aimed to provide a general overview of the recent advancement and the hotspots in the field of stroke and astrocyte from 2001 to 2021.

**Materials and methods:**

Publications between 2001 and 2021, related to stroke and astrocyte were retrieved from the Web of Science (WOS) and analyzed in Gephi and VOSviewer.

**Results:**

In total, 3789 documents were extracted from the WOS databases. The publications showed stable growth since 2001. The United States and China were the most prolific countries and University of California San Francisco and Oakland University were the most influential institutes. The top four most productive journals were Brain Research, Journal of Cerebral Blood Flow and Metabolism, Glia and Journal of Neuroinflammation. Keywords frequency and co-occurrence analysis revealed that the topics related to “micro-RNA”, “toll like receptor”, “neuroinflammation”, “autophagy” and “interleukin” were research frontiers. The field of stroke and astrocyte focused on several aspects, such as the role of astrocytes in the treatment of stroke, metabolic changes in astrocytes, the protective role of apoptosis in astrocytes after oxidative stress injury and neurovascular units.

**Conclusion:**

This comprehensive bibliometric study provides an updated perspective on the trend of research associated with stroke and astrocyte. It will benefit scientific community to identify the important issues, future directions and provide a novel understanding of stroke pathophysiology, hotspots and frontiers to facilitate future research direction.

## Introduction

Stroke, broadly classified into ischemic stroke and hemorrhagic stroke, has been a serious public health problem worldwide. Several studies report that stroke is the second leading cause of death and major contributor of disability-adjusted life years (DALYs). In 2019, the global incidence of stroke events was around 101.47 million with 12.2 million new strokes (Collaborators, 2021). It is expected that there will be more than 200 million stroke survivors, almost 300 million DALYs, 25 million new strokes, and 13 million deaths from stroke annually by 2050 (Collaborators, 2021). It has been shown that the absolute numbers of stroke survivors have increased globally, partly due to rising numbers in low- and middle-income countries (Katan and Luft, 2018). Complex interwoven issues of inaccessibility to therapeutic management and interventions limit the effectiveness of primary and secondary prevention in stroke care (Kim et al., 2020). Abundant medical resource requirements for stroke treatment and follow-up management increase the financial burden on patients’ families and healthcare systems.

Astrocytes are important component of neurovascular unit (NVU) structure composition and play a vital role in the regulation of central nervous system (CNS) functions. The onset of stroke activates astrocytes and contributes to differentiating astrocytes into several phenotypes. Neuroinflammation and neuroprotection effects in stroke are due to the activation of astrocytes into “A1s” and “A2s” (Zamanian et al., 2012). In acute phase of stroke, A1 reactive astrocytes predominate and secrete inflammatory factors that exacerbate neuronal and blood-brain barrier (BBB) damage and form glial scar to limit the spread of inflammation (Xu et al., 2020). Conversely, in the subacute and chronic phases, A2 reactive astrocytes play a protective role by secreting neurotrophic factors to promote neurogenesis, synaptogenesis (Yamagata, 2021), angiogenesis (Williamson et al., 2021), and BBB repair (Song et al., 2021). However, the categorization of reactive astrocytes in pathological conditions is controversial, and the understanding of astrocytic heterogeneity in response to ischemic stroke is limited and still needs further discussion (Li et al., 2022; Ma et al., 2022). Extensive research on the pathological mechanisms, diagnosis, treatment, and prognosis of stroke has been conducted to provide new research perspectives in recent decades (Kuriakose and Xiao, 2020). Therefore, we have summarized the key findings and trends of stroke research in two decades (2001 to 2021) (Supplementary Table 1). We showed an overview of the mechanisms, functions, and potential therapeutic targets of astrocytes involved in the development of stroke. It is expected to help the scientific community to explore new research trends, track research hotspots and identify the novel research direction in future.

Bibliometric analysis is an effective tool used for mapping and identifying the published records. Currently, it has been widely recognized as an alternative method to evaluate the hot issues in several fields of neuroscience (Zhang et al., 2021; Miao et al., 2022). The methods included co-authorship network, co-occurrence of author keywords, highly cited paper analysis, impact factor and h-index. Therefore, comprehensive bibliometric methods and indicators were used to have a deeper insight into the research trends, the most concerned topics, and existing research gaps in the field of stroke and astrocyte. Our study showed a systematic and comprehensive analysis of the most contributing factors and development trends in this field through bibliometric analysis.

## Materials and methods

### Data source

The data in this study were extracted from the Web of Science (WOS) core collection, including the Science Citation Index-expanded (SCI-E), and Social Sciences Citation Index (SSCI). The WOS database provides comprehensive, multi-disciplinary citation data, and served as the major source of data for bibliometric analysis. The SCI-E and SSCI databases are the most frequently used databases for bibliometric studies (Peng et al., 2015). We conducted a comprehensive online search in WOS database from 2001 to 2021, with restricted document types to original articles and reviews. The searched items are shown in Supplementary Table 2. A total of 3789 published articles were retrieved and each contains author names, institutions, titles, abstracts, total citations, keywords, sources and cited references.

The keywords provided by the authors were considered in this study. Firstly, synonym terms were manually merged, e.g., “brain ischemia” and “cerebral ischemia”. Secondly, to improve the visualization, acronyms were generally favored, e.g., “GFAP” for “glial fibrillary acidic protein.” Similarly, “oxygen glucose deprivation” was shortened for “OGD.” The singular form and the plural form of the same noun (e.g., Astrocytes and Astrocyte) were grouped.

### Methods

The scientific performance of an entity (e.g., an institution, a country or a journal) was measured on the basis of productivity and quality of research publications. We adopted the following four indicators in this study. The first indicator concerns the output. The second and third indicators focus on quality and the fourth indicator accounts for paper output and the citation impact:

(1) Total publications (TP): Total number of publications of an entity during the observation period.
(2) Total citations (TC): Total number of citations of articles published by an entity during the observation period.
(3) TC/TP: Average citation per publication.
(4) H-index served to reflect the academic influence of an institution, a country or a journal. The index of H means that an institution, a country or a journal has published H publications and each of which has been cited by other papers at least H times (Hirsch and Buela-Casal, 2014).

VOSviewer is an optimal software tool to analyze the research collaboration among institutions. It is also used to map and visualize the network of keywords. VOSviewer can classify keywords into different clusters through co-occurrence analysis, and simultaneously color them by time course. Gephi is the leading visualization and exploratory software for network analysis. In this study, Gephi software was used to visualize the collaboration network among countries (Bastian et al., 2009). CitNetExplorer is a software tool for visualization and analysis of citation networks among scientific publications (Eck and Waltman, 2014). It was used to identify the critical findings in stroke and astrocyte research.

## Results

### General statistics

**FIGURE 1:**
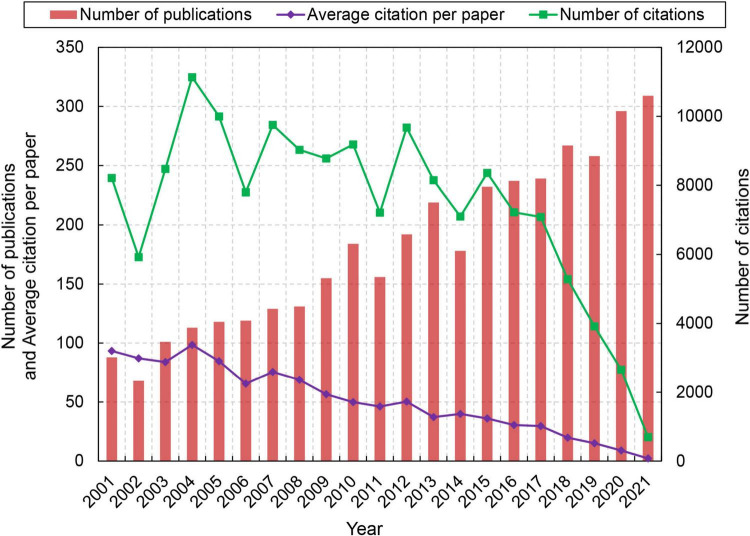
Trend in the number of publications in the field of stroke and astrocyte during the period 2001–2021.

Figure 1 shows the trend of publications related to stroke and astrocyte. The number of publications on stroke and astrocyte increased steadily during the past 20 years. The paper output increased by 251.1% (from 88 in 2001 to 309 in 2021), indicating that researchers are paying more attention to this field. The total citation frequency showed significant fluctuation before 2015, reached the peak of 11,134 in 2004, and declined steadily from 2015 to 2021. The average citation per paper significantly decreased by 97.6% (from 93.32 in 2001 to 2.24 in 2021).

### Most productive and influential countries

From 2001 to 2021, 70 countries contributed to stroke and astrocyte research by publishing academic papers in WOS database. However, 95.51% of publications were contributed by the top 20 countries as shown in Table 1. The United States published the highest number of papers (1191), accounting for 31.43%, followed by China (1108 papers, 29.24%), Japan (416 papers, 10.98%), Germany (287 papers, 7.57%), and South Korea (258 papers, 6.81%). The United States had the highest citation frequency (73,986), which is 2.91 times of China. Sweden had the highest average citation frequency (82.31 citations per paper), followed by Switzerland (72.27), and the United Kingdom (65.76).

**TABLE 1:** Top 20 most productive countries in the field of stroke and astrocyte.

Rank	Country	TP	Percentage (%)	TC	ACPP	H-index
1	United States	1191	31.43	73986	62.12	129
2	China	1108	29.24	25457	22.98	66
3	Japan	416	10.98	15171	36.47	67
4	Germany	287	7.57	14211	49.52	66
5	South Korea	258	6.81	9108	35.3	48
6	Canada	167	4.41	8161	48.87	50
7	United Kingdom	164	4.33	10785	65.76	58
8	Spain	116	3.06	5517	47.56	40
9	Italy	106	2.8	5122	48.32	40
10	Australia	105	2.77	4571	43.53	33
11	Sweden	104	2.74	8560	82.31	45
12	France	96	2.53	3710	38.65	34
13	Brazil	61	1.61	1505	24.67	21
14	Switzerland	60	1.58	4336	72.27	31
15	Poland	57	1.5	1477	25.91	21
16	Netherlands	51	1.35	2584	50.67	26
17	Russia	45	1.19	1353	30.07	13
18	Denmark	38	1	1277	33.61	18
19	Finland	34	0.9	1866	54.88	24
20	Hungary	30	0.79	1123	37.43	14

TP, total number of publications; TC, total citations; ACPP, average citation per paper.

**FIGURE 2:**
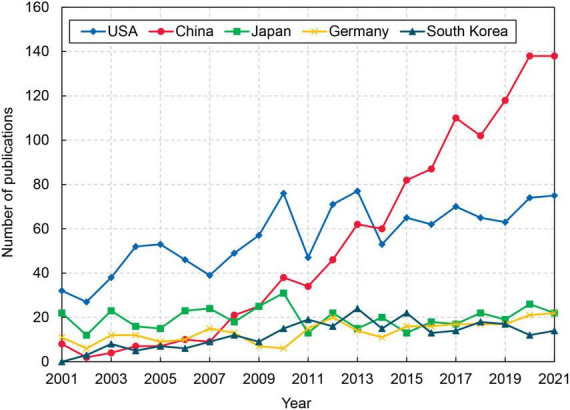
The annual number of top 5 productive countries during 2001–2021.

Figure 2 shows the trend of the top five prolific countries. Studies on astrocytes for stroke in the United States started relatively early. Before 2013, the number of articles published by the United States was higher than other countries. After a sudden decline in 2014, the number of articles began to increase steadily to 75 in 2021. China ranked second in total number of publications related to stroke and astrocyte and growing steadily. It has reached 138 in 2021 after surpassing the United States in 2014. Compared with the United States and China, the growth trend of papers in Japan, Germany and South Korea is not significant, and the number of papers has been at a low level.

**FIGURE 3:**
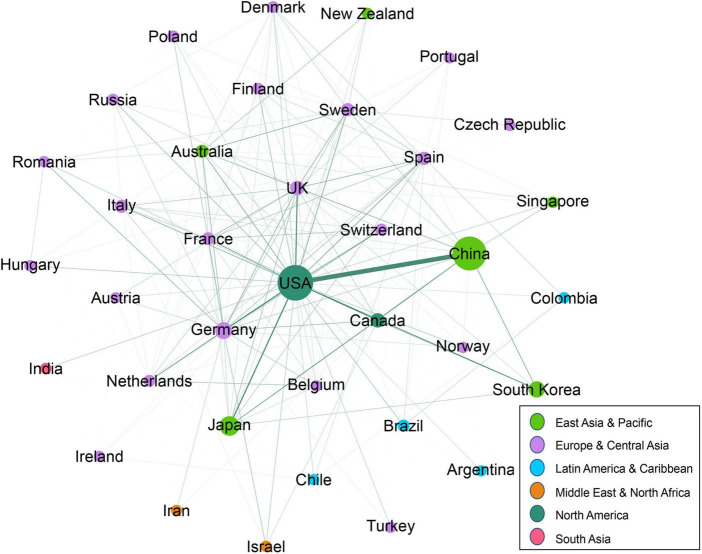
Collaboration networks of the countries with ten or more papers.

In order to explore the characteristics of collaboration among countries in the field of stroke and astrocyte, a cross-border collaboration network was established by including countries with more than 10 publications. In the network, nodes represent countries and connections indicate collaborative relationships. Nodes size represents paper output, and color represents the region. Figure 3 showed that the United States is at the center of the collaborative network with 34 different collaboration partners (Supplementary Figure 1). It is noticeable that among 36 different countries in the collaboration network, 21 from Europe and Central Asia, 6 from East Asia and Pacific, and a few from other regions. Among the network, the United States and China have the closest collaborative relationship, publishing 200 papers together, much higher than those of the second and third ranking of the United States-Japan (48) and the United States-United Kingdom (44), as presented in Supplementary Table 3.

### Most productive and influential institutions

We further identified the most productive and influential institutions in the field of stroke and astrocyte, as shown in Table 2. Seoul National University and Kangwon National University ranked first and second with 62 and 60 papers respectively, followed by Stanford University with 59 papers. In terms of total citations, University of California San Francisco ranked first with 5,727 citations, followed by Oakland University with 5,145 citations. For average citation per article, University of California San Francisco and Oakland University also ranked the top two positions, with an average citation per article of 119.31 and 111.85 respectively. Regarding H index, the University of California San Francisco ranked first with an H index of 35, followed by Oakland University and Stanford University with 32. In general, among the top 20 institutions, 8 from the United States, 5 from China, 4 from South Korea, 2 from Japan, and 1 from Sweden.

**TABLE 2:** Top20 most productive institutes in the field of stroke and astrocyte.

Rank	Institution	Country	TP	TC	ACPP	H-index
1	Seoul National University	South Korea	62	2075	33.47	25
2	Kangwon National University	South Korea	60	991	16.52	18
3	Stanford University	United States	59	4642	78.68	32
4	Hallym University	South Korea	57	1118	19.61	19
5	Massachusetts General Hospital	United States	56	3228	57.64	31
6	University of California San Francisco	United States	48	5727	119.31	35
7	Oakland University	United States	46	5145	111.85	32
8	Loma Linda University	United States	44	1570	35.68	24
9	University of Gothenburg	Sweden	41	4116	100.39	26
10	Fudan University Huashan Hospital	China	39	1135	29.1	18
11	Tongji Hospital, Huazhong University of Science and Technology	China	39	1287	33	22
12	Shanghai Jiao Tong University	China	38	1273	33.5	17
13	Harvard University	United States	37	3423	92.51	31
14	Kyoto University	Japan	37	1473	39.81	20
15	Zhejiang University	China	36	1196	33.22	23
16	Johns Hopkins University	United States	35	2765	79	26
17	Ruijin Hospital Shanghai Jiao Tong University	China	35	1301	37.17	17
18	Okayama University	Japan	33	1588	48.12	18
19	Emory University	United States	33	1765	53.48	21
20	Yonsei University	South Korea	32	2274	71.06	19

TP, total number of publications; TC, total citations; ACPP, average citation per paper.

**FIGURE 4:**
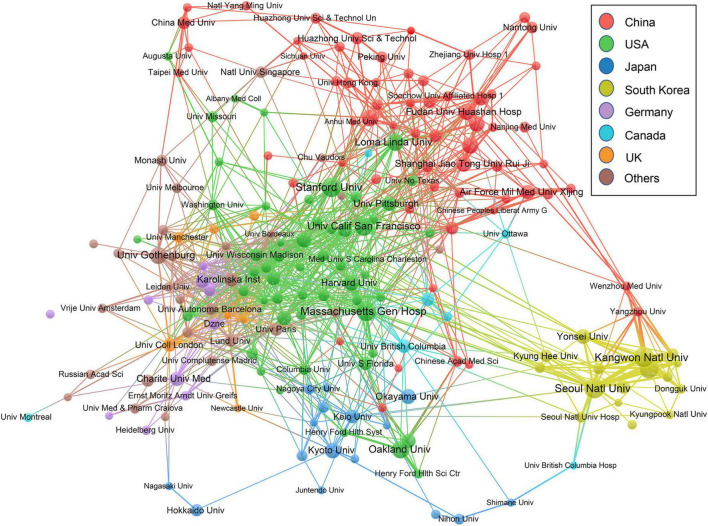
Collaboration networks of the institutions with ten or more papers.

The collaborative relationships among the top 200 productive institutions were also analyzed. As shown in Figure 4, Hallym University and Kangwon National University, both from South Korea, have a strong collaborative relationship. They have published 48 co-authored papers, as shown in Supplementary Table 4. It is worth noting that most closed collaboration relationships are among South Korean institutions. Massachusetts General Hospital located in the United States is at the center of the collaboration network, having collaboration relationships with other 33 institutes, especially with Harvard University, United States. These two United States institutes have produced 22 co-authored publications. The German Center for Neurodegenerative Diseases and University of Washington also have close collaborative relationships with other 32 institutes, as shown in Supplementary Table 5. Within the network, 62 (31%) and 54 (27%) institutions are from China and United States, respectively.

### Analysis of core journals

With respect to source journals, 3,789 articles of our sample have been published in 710 journals. Among 710 journals, 349 journals published only one paper. The top 20 most productive journals in the research domain of stroke and astrocyte, account for 39.61% of the total published articles in our dataset (1,501 out of 3,789) as presented in Table 3. Based on our results, Brain Research is the most productive journal with 146 articles and plays a significant role in the domain. The second and third most productive journals are Journal of Cerebral Blood Flow and Metabolism and Glia with 145 and 102 publications, respectively. In terms of total citation frequency and average citation per paper, Journal of Cerebral Blood Flow, Metabolism, Stroke and Glia ranked relatively high. In addition, 13 of the top 20 productive journals have an impact factor greater than 5. The top 20 prolific journals are mainly distributed in neuroscience discipline.

**TABLE 3:** Top 20 most productive journals in research on stroke and astrocyte.

Rank	Journal	TP	TC	ACPP	H	IF (2020)	WOS category (rank/total number of journals in WOS category)
1	Brain Research	146	5475	37.5	40	3.252	Neurosciences (170/273)
2	Journal of Cerebral Blood Flow and Metabolism	145	11762	81.12	62	6.2	Endocrinology and Metabolism (24/146) Hematology (16/76) Neurosciences (50/273)
3	Glia	102	6850	67.16	47	7.452	Neurosciences (33/273)
4	Journal of Neuroinflammation	96	3061	31.89	34	8.322	Immunology (21/162) Neurosciences (23/273)
5	Stroke	93	7674	82.52	54	7.914	Clinical Neurology (16/208) Peripheral Vascular Disease (6/65)
6	Neuroscience	92	3243	35.25	34	3.59	Neurosciences (141/273)
7	PLoS One	84	3199	38.08	35	3.24	Multidisciplinary Sciences (26/72)
8	Neuroscience Letters	80	2457	30.71	28	3.046	Neurosciences (186/273)
9	Journal of Neurochemistry	75	3423	45.64	34	5.372	Biochemistry and Molecular Biology (78/295) Neurosciences (67/273)
10	Neurochemical Research	73	2195	30.07	21	3.996	Biochemistry and Molecular Biology ( 139/295) Neurosciences (118/273)
11	Journal of Neuroscience Research	65	2574	39.6	29	4.164	Neurosciences (105/273)
12	Neurochemistry International	61	2238	36.69	26	3.921	Biochemistry and Molecular Biology (145/295) Neurosciences (122/273)
13	Experimental Neurology	60	3043	50.72	29	5.33	Neurosciences (70/273)
14	International Journal of Molecular Sciences	57	876	15.37	16	5.924	Biochemistry and Molecular Biology (67/295) Chemistry, Multidisciplinary (49/178)
15	Journal of Neuroscience	54	5815	107.69	36	6.167	Neurosciences (52/273)
16	Molecular Neurobiology	50	1170	23.4	21	5.59	Neurosciences (61/273)
17	Frontiers In Cellular Neuroscience	49	906	18.49	15	5.505	Neurosciences (63/273)
18	Neurobiology of Disease	42	3007	71.6	27	5.996	Neurosciences (54/273)
19	Neural Regeneration Research	40	357	8.93	11	5.135	Cell Biology (78/195) Neurosciences (78/273)
20	Translational Stroke Research	37	723	19.54	17	6.829	Clinical Neurology (24/208) Neurosciences (40/273)

TP, total number of publications; TC, total citations; ACPP, average citation per paper; H, H-index of a journal.

### Influential articles analysis

The citation frequency of scientific papers is commonly used to measure their influence on the related research field. The most highly cited articles were analyzed with parameters such as the total citations, average annual citations, and the authors of the articles for 2001–2021. As shown in Table 4, the most highly cited article with 1373 citations is a comprehensive review paper conducted by Ballabh et al. (2004), describing the role of BBB in neurological diseases. Astrocytes as the component of BBB, also participate in the induction of tight junction disorganization and BBB breakdown in hypoxic-ischemic. Pekny and Nilsson (2005) showed the second highest citation (1,122), focused on several functions of astrocytes in acute, postacute, and chronic stroke phases and elucidates its role as novel target in trauma and ischemia of CNS. In the third highest citation (1,089) Zamanian provided transcriptome databases for two subtypes of reactive astrocytes and suggested that reactive astrocytes in ischemia exhibited a protective molecular phenotype (Zamanian et al., 2012).

**TABLE 4:** Top 10 most cited papers in the field of stroke and astrocyte during 2001–2021.

Title	Author	Journal	TC	PY	TC/Y
The blood-brain barrier: an overview - Structure, regulation, and clinical implications	Ballabh, P; Braun, A; Nedergaard, M	Neurobiology of Disease	1373	2004	72.26
Astrocyte activation and reactive gliosis - A new target in stroke?	Pekny, M; Nilsson, M	Glia	1122	2005	62.33
Genomic Analysis of Reactive Astrogliosis	Zamanian, Jl; Xu, Lj; Foo, Lc, etc.	Journal of Neuroscience	1089	2012	99
Intravenous administration of human umbilical cord blood reduces behavioral deficits after stroke in rats	Chen, Jl; Sanberg, Pr; Li, etc.	Stroke	894	2001	40.64
Directed migration of neural stem cells to sites of CNS injury by the stromal cell-derived factor 1 alpha/CXC chemokine receptor 4 pathway	Imitola, J; Raddassi, K; Park, Ki, etc.	Proceedings of the National Academy of Sciences	838	2004	44.11
Apolipoprotein E controls cerebrovascular integrity *via* cyclophilin A	Bell, Rd; Winkler, Ea; Singh, I, etc.	Nature	738	2012	67.09
Astrocytes and brain injury	Chen, Ym; Swanson, Ra	Journal of Cerebral Blood Flow and Metabolism	685	2003	34.25
The Neurovascular Unit Coming of Age: A Journey through Neurovascular Coupling in Health and Disease	Iadecola, C	Neuron	661	2017	110.2
The Glymphatic System: A Beginner’s Guide	Jessen, Na; Munk, Asf; Lundgaard, I, etc.	Neurochemical Research	651	2015	81.38
Human bone marrow stem cells exhibit neural phenotypes and ameliorate neurological deficits after grafting into the ischemic brain of rats	Zhao, Lr; Duan, Wm; Reyes, M, etc.	Experimental Neurology	640	2002	30.48

TC, total citations; TC/Y, average citation per year.

### Analysis of keywords frequency and co-occurrence network

The underlying justification for performing keywords frequency and co-occurrence analysis is that authors’ keywords effectively represent the main topic and broader scope of articles and their themes (Agnusdei and Coluccia, 2022). Table 5 lists the top 30 highest frequently used keywords. The hot topics related to stroke and astrocyte were astrocyte reactivity including inflammatory response of astrocytes, neuroprotection, apoptosis, oxidative stress, and homeostasis of neurovascular units. It suggests that reactive astrocyte plays an important role in the pathogenesis of stroke. *In vivo* model, MCAO (Middle Cerebral Artery Occlusion) is frequently used to mimic the human ischemic stroke and displays similar penumbra (Kuriakose and Xiao, 2020), while *in vitro* model, OGD (Oxygen-Glucose Deprivation) allows the investigation of specific basic biochemical and molecular mechanisms under conditions of energy deficiency similar to ischemia (Sommer, 2017). Both models showed rapid growth in the frequency of publications in the last 20 years.

**TABLE 5:** Frequency of top 30 most used author keywords in each period.

Keyword	Frequency			
	All years	2001–2007	2008–2014	2015–2021
Astrocyte	1,484	280	508	696
Cerebral ischemia	848	193	294	361
Stroke	766	138	271	357
Ischemic stroke	433	27	109	297
Microglia	391	66	135	190
Inflammation	353	44	112	197
Neuron	339	71	128	140
Neuroprotection	336	57	106	173
Ischemia	330	96	124	110
Brain Injury	319	54	105	160
Blood brain barrier	311	44	94	173
Rat	288	81	101	106
Middle cerebral artery occlusion	249	49	92	108
Mouse	229	50	82	97
Neuroinflammation	221	9	37	175
Apoptosis	218	47	74	97
Reperfusion	204	29	50	125
Therapy	200	34	66	100
Brain	196	39	74	83
GFAP	182	51	72	59
Intracerebral hemorrhage	174	21	49	104
Oxidative stress	161	30	44	87
OGD	160	10	46	104
Dementia	156	36	42	78
Neurogenesis	139	23	53	63
Hippocampus	139	30	55	54
Neurodegenerative disease	138	39	40	59
Neurovascular unit	133	7	36	90
Brain endothelial cell	119	17	53	49
CNS	116	31	47	38

**FIGURE 5:**
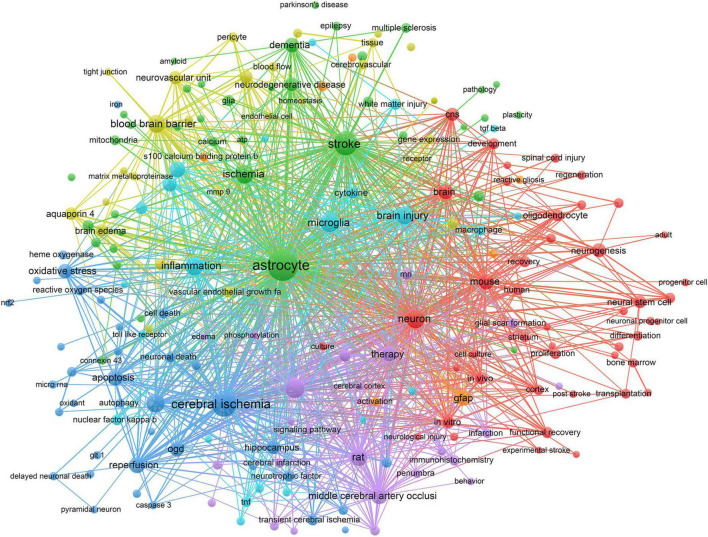
The occurrence network of the most frequently used authors keywords (frequency >20).

A total of 6,704 keywords were extracted from our dataset, and Figure 5 presents the co-occurrence network of 201 keywords with a frequency of 20 and above. For research topic analysis, all the keywords were grouped into 7 major clusters. The first and biggest cluster (red color) containing the most keywords (45) focused on the role of astrocytes in the treatment of stroke, including neural cell development, proliferation, differentiation, regeneration, clinical and basic research in human, animal, and cellular levels. Cluster 2 (green) refers to metabolic changes in astrocytes, including energy metabolism, glutamate metabolism, lactic acid metabolism, etc. Cluster 3 (blue) involves the protective role of apoptosis in astrocytes after oxidative stress injury, including neurotrophic factor secretion, oxidative stress, autophagy, cell death, apoptosis and signaling pathway-related protein kinases. Cluster 4 (yellow) focuses on neurovascular units, including cerebrovascular, cerebral blood flow, BBB, endothelial cells, tight junctions and central homeostasis. Cluster 5 (purple), cluster 6 (light blue), and cluster 7 (orange) represent the assessment of cerebral infarction, neuroinflammation, and reactive astrocyte biomarkers, respectively.

**FIGURE 6:**
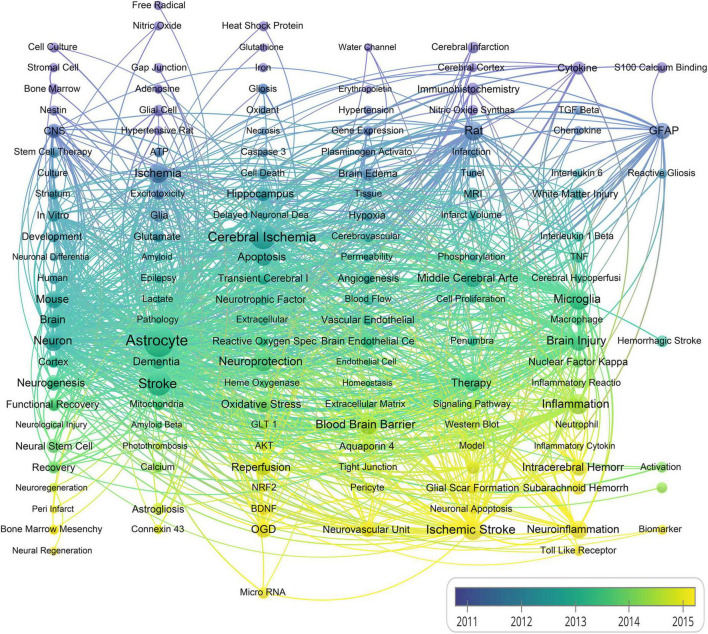
A chronological overview of co-occurrence map of author keywords based on average publication date (The size of the nodes indicates the frequency of the keywords, and the line width between two nodes presents the number of co-occurrence between them).

Next, we calculated the average occurrence of keywords per year. As shown in Figure 6, the keywords colored from blue to green, and further to yellow denote that the average per year is diminishing. Few keywords such as micro-RNA, toll like receptor, neuroinflammation, autophagy and interleukin with a yellow color indicate research hotspots in recent years in the field of stroke and astrocyte.

## Discussion

### The general characteristics of the papers

This bibliometric analysis thoroughly examines the research trends and advancement in the field of stroke and astrocyte from 2001 to 2021 by using social network analysis involving country, institution and author keywords, which provides a comprehensive description of the field for the first time. This temporal analysis revealed that the scientific output in the field of stroke and astrocyte showed substantial growth in publications. At the global level, the United States has been taking a leadership position in stroke and astrocyte research with the largest number of publications as well as the greatest academic influence in this field. Notably, China exceeded the United States in the number of publications since 2014. At the institutional level, the Seoul National University was the most productive institution, followed by Kangwon National University, Stanford University and Hallym University. The top 20 journals contributed 39.61% of total publications related to stroke and astrocyte. Brain Research was the most productive journal followed by Journal of Cerebral Blood Flow and Metabolism, Glia, Journal of Neuroinflammation and Stroke.

### Astrocytes in therapies

Our results showed that the functional recovery mechanisms of neurological injury attract more attention in the recent decade. Astrocyte has been shown in promoting the development of neurocytes by alleviating cell death and promoting neuroregeneration in ischemic stroke injury. Figure 5 shows that research on stem cell therapy have been popular for more than 10 years. Neural stem cells trigger and amplify the endogenous neural recovery process (Zhang et al., 2019). Bone marrow mesenchymal stem cells (BMMSCs) (Li et al., 2021b) and endothelial progenitor cells (EPCs) (Bayraktutan, 2019) have emerged and gradually became the promising candidates for clinical translations. The transplantation of stem cells through intravenous, intra-arterial, and stereotactic approaches confirms the safety and feasibility in clinical application for the treatment of ischemic stroke (Kawabori et al., 2020; Yang et al., 2020).

### Astrocytes in substance-energy metabolism

Astrocytic gap junctions and hemichannels facilitate metabolites exchange and information communication with neurons and oligodendrocytes and maintain intracellular and extracellular homeostasis. Changes in astrocyte substance-energy metabolism indirectly reflect neuronal energy supply. Connexins and pannexins in cerebral ischemia are involved in excitotoxicity, BBB disruption and neuroinflammatory response (Kim et al., 2018). Ischemic stroke decreases cerebral perfusion and reduces the production of ATP, which severely changes neuronal energy homeostasis. Explosive release of excitatory amino acids (such as glutamate) induces calcium influx and thus triggers the release of ROS, NO, and inflammatory factors. These factors may cross the damaged neurons to healthy astrocytes *via* Cx43/Cx36, a major component of astrocyte gap junctions, reducing the load of neurons and activating astrocytes (Liang et al., 2020). However, the mechanism of ischemia induced by astrocytic Cx43 expression remains controversial.

### Astrocytes in oxidative stress

After stroke, astrocytes and microglia release inflammatory factors and ROS through the activation of NF-κB and NADPH oxidase pathways, causing damage to glial cells and neurons (Zhu et al., 2022). Research on nucleic acid therapy bursting (Henninger and Mayasi, 2019) suggests that miRNAs regulate cellular pathways and mitochondrial function in stroke (Jiang et al., 2021; Li et al., 2021a; Halurkar et al., 2022). It provides an efficient and precise method to control these pathways. In addition, various neurotrophic factors secreted by astrocytes, such as nerve growth factor (NGF), brain-derived growth factor (BDNF), erythropoietin (EPO), vascular endothelial growth factor (VEGF), and glial derived neurotrophic factor (GDNF), are increased after stroke (Linnerbauer and Rothhammer, 2020).

### Astrocytes in neurovascular unit

Neurovascular unit consists neuron-glia-vasculature and plays an important role in maintaining the normal physiological function of neurons. After stroke, AQP4 on the surface of astrocytes cause astrocyte swelling and compression of blood vessels, disrupting the BBB integrity and neurovascular unit homeostasis (Ji et al., 2021). Studies have reported that excessive VEGF reduces the expression of tight junctions in endothelial cells, thereby exacerbating BBB injury and neurological deficits (Wen et al., 2017). Furthermore, astrocyte-derived VEGF accelerates the infiltration of peripheral immune cells into the CNS by increasing the expression of ICAM-1 and VCAM-1 in endothelial cells (Moon et al., 2021; Qiu et al., 2021). Conversely, astrocyte-secreted angiopoietin 1 promotes endothelial cell proliferation and BBB formation, thereby improving post-stroke revascularization, enhancing endogenous angiogenesis and promoting neurological functional recovery after cerebral ischemia (Patabendige et al., 2021). Therefore, astrocytes act as mediators of angiogenesis and may provide therapeutic targets for the development of novel neuroprosthetic therapies for stroke.

### Assessment method, glial scar formation, neuroinflammation, biomarkers in stroke

We observed an interesting finding, where MRI was a vital diagnostic tool and assessment method shown in Figure 5. Diffusion-weighted MRI allows earlier detection for clinical intervention as compared to conventional CT diagnosis (Bhat et al., 2021). MRI offers accurate and precise estimation of cellular level neuroenergetic abnormality. Metabolic MRI monitors the process of astrogliosis and its correlation with the neuronal connective pathway over a period (Hao et al., 2016; Alambyan et al., 2020). Laboratory methods assess the reactive astrocytes proliferation, glial scar formation for monitoring subtle changes during pathogenesis.

Reactive astrocytes microglia aggregate to form the glial scars and increase the inhibitory environment by secreting extracellular matrix proteins (such as chondroitin sulfate proteoglycans) into the peri-infarct region (Dzyubenko et al., 2018; Pekny et al., 2019). Although glial scars limit tissue damage during the peak of inflammation and act as the major obstacle for axonal growth during cerebral ischemic stroke recovery. Whether astrocyte scar formation aids axon regeneration needs further exploration (Bradbury and Burnside, 2019). Interestingly, Shi et al. (2021) demonstrated that reactive microgliosis and astrogliosis hinder brain repair by engulfing synapses, and specific inhibition of this phagocytosis before glial scar maturation has beneficial consequences. Several signaling pathways and targets related to astrogliosis and glial scar formation have also been identified (Zhang et al., 2018; Zhu et al., 2021), which indicates that astrocytes and glial scars have great research prospects. In addition to glial scars, the function and role of fibrotic scars, which contains a rich deposit of extracellular matrix proteins, in stroke also deserves our study and attention (Dzyubenko et al., 2018).

Macrophages/microglia are major source of inflammatory mediators (e.g., TGF-β, IL-6, IL-1β, or TNF-α) in stroke to activate the astrocyte TLR/NF-κB inflammatory pathway and further amplify neuroinflammation to exacerbate neuronal damage and stroke symptoms (Leitner et al., 2019). However, microglia and astrocytes play a significant role in maintaining the brain’s innate immune system. Activated microglias extensively communicate with other nerve cells such as astrocytes, and inflammatory crosstalk between cells further aggravates CNS injury (Qiu et al., 2021). The astrocytes in microglial co-culture have been reported to exhibit A2 phenotype and activated to A1 astrocytes by TNF-α and IFN-γ under the stroke-mimicking condition (Kim and Son, 2021). Microglia and astrocytes orchestrate a cascade of immune networks to amplify the crosstalk and interaction in the NVU. Kumar (2019) reported the importance of TLR in the pathology of stroke inflammation by describing the localization of inflammatory mediators in cellular signaling pathways. These findings suggest that regulating the activation of microglia could be potential therapeutic strategy to intervene the polarization switching of astrocytes.

Reactive astrocyte proliferation is characterized by GFAP and S100β and serves as vital biomarker (Ding et al., 2021). Increased GFAP serum level corresponds with the higher value for NIHSS and correlated with stroke severity and the extent of brain damage in ischemic stroke patients (Amalia, 2021). The astroglial protein S100B serum concentrations are measured at 48–72 h after symptom onset and are highly correlated to final infarct volume and functional outcome (Luger et al., 2021). miRNA expression in astrocytes is stable and reported to be secreted by astrocytic exosomes together with circular RNA (circRNA) (Chen et al., 2020; Pei et al., 2020) to inhibit OGD-induced autophagy and neuronal apoptosis. The concentration of miRNAs (Mirzaei et al., 2018) and circRNA (Han et al., 2018) in the blood crossing from the BBB could be potential biomarker for stroke prognosis and potentially predict the outcome of post-stroke rehabilitation.

## Limitations

The purpose of this study is to summarize the literature in the field of stroke and astrocyte, projects the recent trends and research hotspots. However, the major limitation of this study is inclusion of only WOS database, while other databases such as Scopus or PubMed have not been considered. Multiple databases could be included in the future study to verify the robustness of this study. Also, this study involved only articles and reviews. The analyses of different types of publications, including book chapters, conference proceedings and other documental types may be considered in future studies.

## Conclusion

In this study, we provided a panorama-to-profile up-to-date analysis on the research trends and hot issues of stroke and astrocyte, based on the bibliometric analysis of publications from 2001 to 2021. Statistical results showed significantly increased concern of stroke and astrocyte in the past two decades and China contributed 50% increase in the last seven years. The increased publications in other countries are relatively slow. The United States and China were the most productive and influential countries with the highest number of total citations, high publications and the most collaborative countries. The University of California San Francisco has a significant academic influence in the field. The research issues can be categorized into seven aspects: (1) the role of astrocytes in the treatment of stroke; (2) metabolic changes in astrocytes; (3) the protective role of apoptosis in astrocytes after oxidative stress injury; (4) neurovascular units; (5) the assessment of cerebral infarction; (6) neuroinflammation; and (7) reactive astrocyte biomarkers. Items such as Micro-RNA, TLR, neuroinflammation, autophagy and interleukin related topic were the emerging research hotspots.

## Data availability statement

The raw data supporting the conclusions of this article will be made available by the authors, without undue reservation.

## Author contributions

ZD and YS: conceptualization. ZD, NJ, TY, HH, YW, LS, and YS: data collection and analysis. ZD, XL, and YS: writing—original draft preparation. ZD, GK, YS, and CM: writing—review and editing. YS: visualization. All authors contributed to the article and approved the submitted version.

## References

[B1] Agnusdei G. P. Coluccia B. (2022). Sustainable agrifood supply chains: Bibliometric, network and content analyses. *Sci. Total Environ.* 824:153704. 10.1016/j.scitotenv.2022.153704 35134421

[B2] Alambyan V. Pace J. Sukpornchairak P. Yu X. Alnimir H. Tatton R. (2020). Imaging guidance for therapeutic delivery: The dawn of neuroenergetics. *Neurotherapeutics* 17 522–538. 10.1007/s13311-020-00843-4 32240530 PMC7283376

[B3] Amalia L. (2021). Glial Fibrillary Acidic Protein (GFAP): Neuroinflammation biomarker in acute ischemic stroke. *J. Inflamm. Res.* 14 7501–7506. 10.2147/JIR.S342097 35002283 PMC8722682

[B4] Ballabh P. Braun A. Nedergaard M. (2004). The blood-brain barrier: An overview: Structure, regulation, and clinical implications. *Neurobiol. Dis.* 16 1–13. 10.1016/j.nbd.2003.12.016 15207256

[B5] Bastian M. Heymann S. Jacomy M. (2009). “Gephi: An open source software for exploring and manipulating networks,” in *Proceedings of the Third International Conference on Weblogs and Social Media, ICWSM 2009*, (San Jose, CA: The AAAI Press).

[B6] Bayraktutan U. (2019). Endothelial progenitor cells: Potential novel therapeutics for ischaemic stroke. *Pharmacol. Res.* 144 181–191. 10.1016/j.phrs.2019.04.017 31004788

[B7] Bhat S. S. Fernandes T. T. Poojar P. Ferreira M. D. Rao P. C. Hanumantharaju M. C. (2021). Low-field MRI of Stroke: Challenges and opportunities. *J. Magn. Reson. Imaging* 54 372–390. 10.1002/jmri.27324 32827173

[B8] Bradbury E. J. Burnside E. R. (2019). Moving beyond the glial scar for spinal cord repair. *Nat. Commun.* 10:3879. 10.1038/s41467-019-11707-7 31462640 PMC6713740

[B9] Chen W. Wang H. Zhu Z. Feng J. Chen L. (2020). Exosome-shuttled circSHOC2 from IPASs regulates neuronal autophagy and ameliorates ischemic brain injury *via* the miR-7670-3p/SIRT1 axis. *Mol. Ther. Nucleic Acids* 22 657–672. 10.1016/j.omtn.2020.09.027 33230464 PMC7581834

[B10] Collaborators G. B. D. S. (2021). Global, regional, and national burden of stroke and its risk factors, 1990-2019: A systematic analysis for the global burden of disease study 2019. *Lancet Neurol.* 20 795–820. 10.1016/S1474-4422(21)00252-0 34487721 PMC8443449

[B11] Ding Z. B. Song L. J. Wang Q. Kumar G. Yan Y. Q. Ma C. G. (2021). Astrocytes: A double-edged sword in neurodegenerative diseases. *Neural. Regen. Res.* 16 1702–1710. 10.4103/1673-5374.306064 33510058 PMC8328766

[B12] Dzyubenko E. Manrique-Castano D. Kleinschnitz C. Faissner A. Hermann D. M. (2018). Role of immune responses for extracellular matrix remodeling in the ischemic brain. *Ther. Adv. Neurol. Disord* 11:1756286418818092. 10.1177/1756286418818092 30619510 PMC6299337

[B13] Eck N. Waltman L. (2014). Citnetexplorer: A new software tool for analyzing and visualizing citation networks. *J. Informetr.* 8 802–823. 10.1016/j.joi.2014.07.006

[B14] Halurkar M. S. Wang J. J. Chen S. Z. Bihl J. C. (2022). EPC-EXs improve astrocyte survival and oxidative stress through different uptaking pathways in diabetic hypoxia condition. *Stem Cell Res. Ther.* 13:91. 10.1186/s13287-022-02766-7 35241178 PMC8896364

[B15] Han B. Zhang Y. Zhang Y. H. Bai Y. Chen X. F. Huang R. R. (2018). Novel insight into circular RNA HECTD1 in astrocyte activation *via* autophagy by targeting MIR142-TIPARP: Implications for cerebral ischemic stroke. *Autophagy* 14 1164–1184. 10.1080/15548627.2018.1458173 29938598 PMC6103660

[B16] Hao X. Z. Yin L. K. Zhang X. X. Tian J. Q. Li C. C. Feng X. Y. (2016). Combining systemic and stereotactic MEMRI to detect the correlation between gliosis and neuronal connective pathway at the chronic stage after stroke. *J. Neuroinflammation* 13:156. 10.1186/s12974-016-0622-7 27316350 PMC4912752

[B17] Henninger N. Mayasi Y. (2019). Nucleic acid therapies for ischemic stroke. *Neurotherapeutics* 16 299–313. 10.1007/s13311-019-00710-x 30635869 PMC6554367

[B18] Hirsch J. E. Buela-Casal G. (2014). The meaning of the h-index. *Int. J. Clin. Health Psychol*. 14, 161–164. 10.1016/s1697-2600(14)70050-x

[B19] Ji C. H. Yu X. Xu W. L. Lenahan C. Tu S. Shao A. W. (2021). The role of glymphatic system in the cerebral edema formation after ischemic stroke. *Exp. Neurol.* 340:113685. 10.1016/j.expneurol.2021.113685 33676917

[B20] Jiang Z. X. Chen J. Chen J. J. Lei Z. L. Chen H. L. Wu J. Q. (2021). Anti-inflammatory effects of paeoniflorin caused by regulation of the hif1a/miR-210/caspase1/GSDMD signaling pathway in astrocytes: A novel strategy for hypoxia-induced brain injury in rats. *Immunopharmacol. Immunotoxicol.* 43 410–418. 10.1080/08923973.2021.1924194 34114917

[B21] Katan M. Luft A. (2018). Global burden of stroke. *Semin. Neurol.* 38 208–211. 10.1055/s-0038-1649503 29791947

[B22] Kawabori M. Shichinohe H. Kuroda S. Houkin K. (2020). Clinical trials of stem cell therapy for cerebral ischemic stroke. *Int. J. Mol. Sci.* 21:7380. 10.3390/ijms21197380 33036265 PMC7582939

[B23] Kim J. Thayabaranathan T. Donnan G. A. Howard G. Howard V. J. Rothwell P. M. (2020). Global stroke statistics 2019. *Int. J. Stroke* 15 819–838. 10.1177/1747493020909545 32146867

[B24] Kim S. Son Y. (2021). Astrocytes stimulate microglial proliferation and M2 polarization *in vitro* through crosstalk between astrocytes and microglia. *Int. J. Mol. Sci.* 22:8800. 10.3390/ijms22168800 34445510 PMC8396240

[B25] Kim Y. Davidson J. O. Green C. R. Nicholson L. F. B. O’Carroll S. J. Zhang J. (2018). Connexins and Pannexins in cerebral ischemia. *Biochim. Biophys. Acta Biomembr.* 1860 224–236. 10.1016/j.bbamem.2017.03.018 28347700

[B26] Kumar V. (2019). Toll-like receptors in the pathogenesis of neuroinflammation. *J. Neuroimmunol.* 332 16–30. 10.1016/j.jneuroim.2019.03.012 30928868

[B27] Kuriakose D. Xiao Z. (2020). Pathophysiology and treatment of stroke: Present status and future perspectives. *Int. J. Mol. Sci.* 21:7609. 10.3390/ijms21207609 33076218 PMC7589849

[B28] Leitner G. R. Wenzel T. J. Marshall N. Gates E. J. Klegeris A. (2019). Targeting toll-like receptor 4 to modulate neuroinflammation in central nervous system disorders. *Exp. Opin. Ther. Targets* 23 865–882. 10.1080/14728222.2019.1676416 31580163

[B29] Li L. Y. Zhou J. P. Han L. Y. Wu X. Shi Y. W. Cui W. X. (2022). The specific role of reactive astrocytes in stroke. *Front. Cell. Neurosci.* 16:850866. 10.3389/fncel.2022.850866 35321205 PMC8934938

[B30] Li W. F. Shi L. L. Hu B. Hong Y. M. Zhang H. Li X. (2021b). Mesenchymal stem cell-based therapy for stroke: Current understanding and challenges. *Front. Cell. Neurosci.* 15:628940. 10.3389/fncel.2021.628940 33633544 PMC7899984

[B31] Li L. Voloboueva L. Griffiths B. B. Xu L. J. Giffard R. G. Stary C. M. (2021a). MicroRNA-338 inhibition protects against focal cerebral ischemia and preserves mitochondrial function *in vitro* in astrocytes and neurons *via* COX4I1. *Mitochondrion* 59 105–112. 10.1016/j.mito.2021.04.013 33933660 PMC8292173

[B32] Liang Z. Wang X. Hao Y. L. Qiu L. Lou Y. Y. Zhang Y. T. (2020). The multifaceted role of astrocyte Connexin 43 in ischemic stroke through forming hemichannels and Gap junctions. *Front. Neurol.* 11:703. 10.3389/fneur.2020.00703 32849190 PMC7411525

[B33] Linnerbauer M. Rothhammer V. (2020). Protective functions of reactive astrocytes following central nervous system insult. *Front. Immunol.* 11:573256. 10.3389/fimmu.2020.573256 33117368 PMC7561408

[B34] Luger S. Koerbel K. Martinez Oeckel A. Schneider H. Maurer C. J. Hintereder G. (2021). Role of S100B serum concentration as a Surrogate outcome parameter after mechanical thrombectomy. *Neurology* 97 e2185–e2194. 10.1212/WNL.0000000000012918 34635559 PMC8641970

[B35] Ma H. Y. Zhou Y. Li Z. F. Zhu L. J. Li H. Zhang G. H. (2022). Single-cell RNA-sequencing analyses revealed heterogeneity and dynamic changes of metabolic pathways in astrocytes at the acute phase of ischemic stroke. *Oxid. Med. Cell. Longev.* 2022:1817721. 10.1155/2022/1817721 35535357 PMC9078813

[B36] Miao H. H. Yu K. Gao D. Y. Lin X. W. Cao Y. Liu X. (2022). A bibliometric analysis of research on Ketamine from 2001 to 2020. *Front. Mol. Neurosci.* 15:839198. 10.3389/fnmol.2022.839198 35283728 PMC8908203

[B37] Mirzaei H. Momeni F. Saadatpour L. Sahebkar A. Goodarzi M. Masoudifar A. (2018). MicroRNA: Relevance to stroke diagnosis, prognosis, and therapy. *J. Cell. Physiol.* 233 856–865. 10.1002/jcp.25787 28067403

[B38] Moon S. Chang M. S. Koh S. H. Choi Y. K. (2021). Repair Mechanisms of the Neurovascular Unit after Ischemic Stroke with a Focus on VEGF. *Int. J. Mol. Sci.* 22:8543. 10.3390/ijms22168543 34445248 PMC8395233

[B39] Patabendige A. Singh A. Jenkins S. Sen J. Chen R. (2021). Astrocyte activation in neurovascular damage and repair following ischaemic stroke. *Int. J. Mol. Sci.* 22:4280. 10.3390/ijms22084280 33924191 PMC8074612

[B40] Pei X. Li Y. Zhu L. Zhou Z. (2020). Astrocyte-derived exosomes transfer miR-190b to inhibit oxygen and glucose deprivation-induced autophagy and neuronal apoptosis. *Cell Cycle* 19 906–917. 10.1080/15384101.2020.1731649 32150490 PMC7217362

[B41] Pekny M. Nilsson M. (2005). Astrocyte activation and reactive gliosis. *Glia* 50 427–434. 10.1002/glia.20207 15846805

[B42] Pekny M. Wilhelmsson U. Tatlisumak T. Pekna M. (2019). Astrocyte activation and reactive gliosis-A new target in stroke? *Neurosci. Lett.* 689 45–55. 10.1016/j.neulet.2018.07.021 30025833

[B43] Peng Y. Lin A. Wang K. Liu F. Zeng F. Yang L. (2015). Global trends in DEM-related research from 1994 to 2013: A bibliometric analysis. *Scientometrics* 105 347–366. 10.1007/s11192-015-1666-7

[B44] Qiu Y. M. Zhang C. L. Chen A. Q. Wang H. L. Zhou Y. F. Li Y. N. (2021). Immune cells in the BBB disruption after acute ischemic stroke: Targets for immune therapy? *Front. Immunol.* 12:678744. 10.3389/fimmu.2021.678744 34248961 PMC8260997

[B45] Shi X. J. Luo L. L. Wang J. X. Shen H. Li Y. F. Mamtilahun M. (2021). Stroke subtype-dependent synapse elimination by reactive gliosis in mice. *Nat. Commun.* 12:6943. 10.1038/s41467-021-27248-x 34836962 PMC8626497

[B46] Sommer C. J. (2017). Ischemic stroke: Experimental models and reality. *Acta Neuropathol.* 133 245–261. 10.1007/s00401-017-1667-0 28064357 PMC5250659

[B47] Song S. S. Huang H. C. Guan X. D. Fiesler V. Bhuiyan M. I. H. Liu R. J. (2021). Activation of endothelial Wnt/beta-catenin signaling by protective astrocytes repairs BBB damage in ischemic stroke. *Prog. Neurobiol.* 199:101963. 10.1016/j.pneurobio.2020.101963 33249091 PMC7925353

[B48] Wen L. J. Tan Y. A. Dai S. H. Zhu Y. Meng T. T. Yang X. Q. (2017). VEGF-mediated tight junctions pathological fenestration enhances doxorubicin-loaded glycolipid-like nanoparticles traversing BBB for glioblastoma-targeting therapy. *Drug Deliv.* 24 1843–1855. 10.1080/10717544.2017.1386731 29182025 PMC8241127

[B49] Williamson M. R. Fuertes C. J. A. Dunn A. K. Drew M. R. Jones T. A. (2021). Reactive astrocytes facilitate vascular repair and remodeling after stroke. *Cell Rep.* 35:109048. 10.1016/j.celrep.2021.109048 33910014 PMC8142687

[B50] Xu S. B. Lu J. N. Shao A. W. Zhang J. H. Zhang J. M. (2020). Glial cells: Role of the immune response in ischemic stroke. *Front. Immunol.* 11:294. 10.3389/fimmu.2020.00294 32174916 PMC7055422

[B51] Yamagata K. (2021). Astrocyte-induced synapse formation and ischemic stroke. *J. Neurosci. Res.* 99 1401–1413. 10.1002/jnr.24807 33604930

[B52] Yang C. Wang X. Tang X. Bao X. Wang R. (2020). Research trends of stem cells in ischemic stroke from 1999 to 2018: A bibliometric analysis. *Clin. Neurol. Neurosurg.* 192:105740. 10.1016/j.clineuro.2020.105740 32114325

[B53] Zamanian J. L. Xu L. J. Foo L. C. Nouri N. Zhou L. Giffard R. G. (2012). Genomic analysis of reactive astrogliosis. *J. Neurosci.* 32 6391–6410. 10.1523/JNEUROSCI.6221-11.2012 22553043 PMC3480225

[B54] Zhang G. L. Zhu Z. H. Wang Y. Z. (2019). Neural stem cell transplantation therapy for brain ischemic stroke: Review and perspectives. *World J. Stem Cells* 11 817–830. 10.4252/wjsc.v11.i10.817 31692854 PMC6828598

[B55] Zhang J. H. Zhang Y. X. Hu L. Y. Huang X. X. Liu Y. F. Li J. Y. (2021). Global trends and performances of magnetic resonance imaging studies on acupuncture: A bibliometric analysis. *Front. Neurosci.* 14:620555. 10.3389/fnins.2020.620555 33551731 PMC7854454

[B56] Zhang R. R. Wu Y. P. Xie F. Zhong Y. L. Wang Y. Xu M. X. (2018). RGMa mediates reactive astrogliosis and glial scar formation through TGF beta 1/Smad2/3 signaling after stroke. *Cell Death Differ.* 25 1503–1516. 10.1038/s41418-018-0058-y 29396549 PMC6113216

[B57] Zhu G. G. Wang X. Y. Chen L. X. Lenahan C. Fu Z. X. Fang Y. J. (2022). Crosstalk between the oxidative stress and glia cells after stroke: From mechanism to therapies. *Front. Immunol.* 13:852416. 10.3389/fimmu.2022.852416 35281064 PMC8913707

[B58] Zhu Y. M. Lin L. Wei C. Guo Y. Qin Y. Li Z. S. (2021). The key regulator of necroptosis, RIP1 kinase, contributes to the formation of astrogliosis and glial scar in ischemic stroke. *Transl. Stroke Res.* 12 991–1017. 10.1007/s12975-021-00888-3 33629276 PMC8557200

