# Supplementary material for: Mapping the research trends of astrocytes in stroke: A bibliometric analysis
Source: Front Cell Neurosci. 2022 Sep 8;16:949521. doi: 10.3389/fncel.2022.949521 (PMC9492963; doi:10.3389/fncel.2022.949521)
Supplement: Supplementary file 1 [file Data_Sheet_1.docx]

Supplementary Material

Supplementary Table 1. Chronology of astrocytes and stroke

| Year(s) | Event |
| --- | --- |
| 1982 | Discovery of early astrocytic proliferation in non-infarcted area after transient cerebral ischemia (Petito and Babiak, 1982) |
| 1993 | Observed three types of astrocytic microscopic changes following middle cerebral-artery occlusion (Garcia et al., 1993) |
| 1993 | Degree of astrocytes proliferation indirectly reflected neuronal injury (Petito et al., 1993) |
| 1995 | Recognition of dual reactivity of astrocyte in focal ischemia (Schroeter et al., 1995) |
| 1996 | Correlation of infarct formation to early expression of glial fibrillary acidic protein (Yamashita et al., 1996) |
| 1998 | Modulation of astrocytes differential response by stroke-induced inflammation (Stoll et al., 1998) |
| 2000-2003 | Targeting molecules of brain edema after stroke: aquaporin-4(AQP4), AQP9 and anchoring of AQP4 to alpha-syntrophin(Manley et al., 2000; Badaut et al., 2001; Amiry-Moghaddam et al., 2003) |
| 2001-2003 | Protective effects of astrocytes gap junction under ischemia (Siushansian et al., 2001; Nakase et al., 2003) |
| 2001 | Human umbilical cord blood cells transplantation as a cell source to treat stroke (Chen et al., 2001) |
| 2002 | First description of S-100beta related to the occurrence of delayed infarct expansion (Matsui et al., 2002) |
| 2002 | Clarification of erythropoietin as a paracrine neuroprotective mediator of ischemic preconditioning (Ruscher et al., 2002) |
| 2003 | First human neural stem cells transplantation improving intracerebral hemorrhage outcome via differentiating into neurons and astrocytes (Jeong et al., 2003) |
| 2003 | Accelerated formation of glial scar in aged rats impeded functional recovery after stroke (Badan et al., 2003) |
| 2005 | Enhancement of neuronal ischemic tolerance via astrocytic glutamate transporter 1(Kawahara et al., 2005) |
| 2005 | Function of reactive gliosis in CNS ischemia (Pekny and Nilsson, 2005) |
| 2007 | Regulation of excessive astrogliosis using cell cycle inhibition attenuated secondary neuronal injury (Zhu et al., 2007) |
| 2008 | Bone marrow stromal cells promoted axonal regeneration by reducing neurocan expression in peri-infarct astrocytes (Shen et al., 2008) |
| 2009 | Effect of ischemia-induced enhancement of astrocytic Ca2+ signal on neuronal damage (Ding et al., 2009) |
| 2009 | AQP facilitated water efflux leading astrocytes to better recover from brief ischemic insult (Risher et al., 2009) |
| 2010-2012 | Proposed role of reactive astrocytes on promoting neurovascular remodeling; e.g., high-mobility group box 1 (Hayakawa et al., 2010; Hayakawa et al., 2012) |
| 2011 | The number of astrocytes proliferating fell sharply with distance to the infarct (Barreto et al., 2011) |
| 2011-2014 | Proliferation,activation and latent neurogenesis of astrocytes are regulated by Notch 1 signal after stroke (Shimada et al., 2011; Magnusson et al., 2014) |
| 2012 | CD36 as a novel mediator for ischemic injury-induced astrogliosis and scar formation (Bao et al., 2012) |
| 2012 | A gene expression profile of populations of reactive astrocytes isolated at various injury time points using GeneChip arrays (Zamanian et al., 2012) |
| 2012 | A newly described membrane-bound astrocyte growth inhibitor ephrin-A5 limited axonal sprouting and motor recovery in stroke (Overman et al., 2012) |
| 2013 | Sonic hedgehog (SHH) directly elicited the stem cell response of astrocyte (Sirko et al., 2013) |
| 2013 | Inositol 1,4,5 trisphosphate-Ca2+ signaling in astrocytes:a key component of endogenous neuroprotection(Zheng et al., 2013) |
| 2013 | Supportive role of intermediate filaments for astrocyte antioxidant stress during oxygen-glucose deprivation and reperfusion (DePablo et al., 2013) |
| 2016 | A new mitochondrial mechanism of neuroglial crosstalk during stroke, involving CD38 and cyclic ADP ribose signaling (Hayakawa et al., 2016) |
| 2016 | Description of astroglial diversity responses to stroke (Pekny et al., 2016) |
| 2019 | Identification of astrocyte-derived factors in BBB function; e.g., nitric oxide, endothelin-1, angiopoietin-1 and SHH (Michinaga and Koyama, 2019) |
| 2019 | Established a stroke-specific astroglial translatome database and identified Stat3, Sp1, and Spi1 as the most prominent transcription factors (Rakers et al., 2019) |
| 2021 | Pyroptosis in astrocytes damaged BBB integrity and glymphatic function and thus influenced A beta clearance (Lyu et al., 2021) |
| 2021 | Therapeutic targets related to glymphatic system and cerebral edema after ischemic stroke (Ji et al., 2021) |

Supplementary Table 2 Search terms of this study

| Search term |  |
| --- | --- |
| #1 | (TI = ((brain OR cerebellar OR cerebral OR cortical OR hemisphere* OR insular OR "internal carotid artery" OR intracerebral OR intracranial OR medullary OR midbrain OR "occipital lobe" OR "parietal lobe" OR pontine OR subarachnoid OR subcortical OR "temporal lobe" OR "vertebrobasilar artery") AND H*emorrhage)) OR (TI = (("anterior choroidal artery" OR brain OR cerebellar OR cerebral OR cortical OR hemisphere* OR encephalopathy OR insular OR "internal carotid artery" OR medullary OR midbrain OR "occipital lobe" OR "parietal lobe" OR pontine OR subcortical OR "temporal lobe" OR "vertebrobasilar artery") AND infarct*)) OR (TI = (stroke* OR "aneurysmal rupture" OR apoplexy OR "brain isch*emia" OR "brain stem isch*emia" OR "brain vascular accident*" OR "cerebral isch*emia" OR "cerebral stem isch*emia" OR "cerebrovascular accident*" OR "cerebrovascular disease*" OR "cerebrovascular disorder*" OR "cerebrovascular event*" OR "isch*emic encephalopathy"))) and (TI = (Astrocyte OR astrocytic OR Astroglia* OR "Astroglial Cell*" ) |
| #2 | (AK = ((brain OR cerebellar OR cerebral OR cortical OR hemisphere* OR insular OR "internal carotid artery" OR intracerebral OR intracranial OR medullary OR midbrain OR "occipital lobe" OR "parietal lobe" OR pontine OR subarachnoid OR subcortical OR "temporal lobe" OR "vertebrobasilar artery") AND H*emorrhage)) OR (AK = (("anterior choroidal artery" OR brain OR cerebellar OR cerebral OR cortical OR hemisphere* OR encephalopathy OR insular OR "internal carotid artery" OR medullary OR midbrain OR "occipital lobe" OR "parietal lobe" OR pontine OR subcortical OR "temporal lobe" OR "vertebrobasilar artery") AND infarct*)) OR (AK = (stroke* OR "aneurysmal rupture" OR apoplexy OR "brain isch*emia" OR "brain stem isch*emia" OR "brain vascular accident*" OR "cerebral isch*emia" OR "cerebral stem isch*emia" OR "cerebrovascular accident*" OR "cerebrovascular disease*" OR "cerebrovascular disorder*" OR "cerebrovascular event*" OR "isch*emic encephalopathy"))) and (AK = (Astrocyte OR astrocytic OR Astroglia* OR "Astroglial Cell*" ) |
| #3 | (AB = ((brain OR cerebellar OR cerebral OR cortical OR hemisphere* OR insular OR "internal carotid artery" OR intracerebral OR intracranial OR medullary OR midbrain OR "occipital lobe" OR "parietal lobe" OR pontine OR subarachnoid OR subcortical OR "temporal lobe" OR "vertebrobasilar artery") AND H*emorrhage)) OR (AB = (("anterior choroidal artery" OR brain OR cerebellar OR cerebral OR cortical OR hemisphere* OR encephalopathy OR insular OR "internal carotid artery" OR medullary OR midbrain OR "occipital lobe" OR "parietal lobe" OR pontine OR subcortical OR "temporal lobe" OR "vertebrobasilar artery") AND infarct*)) OR (AB = (stroke* OR "aneurysmal rupture" OR apoplexy OR "brain isch*emia" OR "brain stem isch*emia" OR "brain vascular accident*" OR "cerebral isch*emia" OR "cerebral stem isch*emia" OR "cerebrovascular accident*" OR "cerebrovascular disease*" OR "cerebrovascular disorder*" OR "cerebrovascular event*" OR "isch*emic encephalopathy"))) and (AB = (Astrocyte OR astrocytic OR Astroglia* OR "Astroglial Cell*" ) |
| #4 | PY = "2001-2021" |
| #5 | Article type="article" and "review" |
| #6 | (#1 OR #2 OR #3) AND #4 AND #5 |


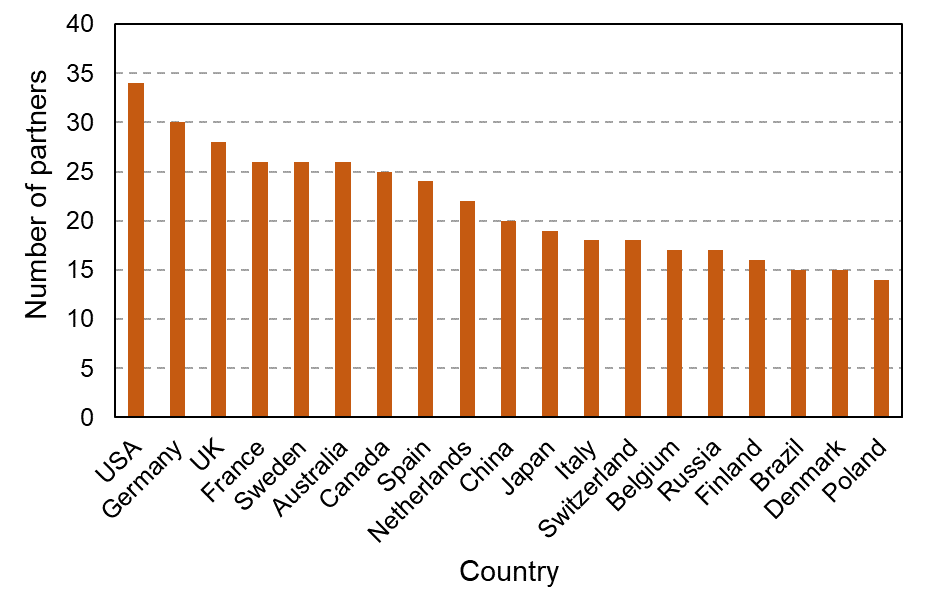


Supplementary Figure 1 The distribution of number of collaboration partners of countries

Supplementary Table 3 Top 18 closest collaboration pair of countries

| Country1 | Country2 | Cooperation frequency |
| --- | --- | --- |
| China | USA | 200 |
| Japan | USA | 48 |
| UK | USA | 44 |
| Germany | USA | 42 |
| Canada | USA | 32 |
| South Korea | USA | 29 |
| France | USA | 18 |
| Sweden | USA | 18 |
| Australia | USA | 17 |
| China | South Korea | 17 |
| Spain | USA | 17 |
| China | Japan | 16 |
| Australia | Sweden | 15 |
| Canada | China | 15 |
| Canada | South Korea | 15 |
| Germany | Netherlands | 15 |
| Germany | UK | 15 |
| Italy | USA | 15 |

Supplementary Table 4 Top 18 closest collaboration pair of institutions

| Institution1 | Institution2 | Cooperation frequency |
| --- | --- | --- |
| Hallym Univ | Kangwon Natl Univ | 48 |
| Kangwon Natl Univ | Seoul Natl Univ | 29 |
| Hallym Univ | Seoul Natl Univ | 28 |
| Henry Ford Hosp | Oakland Univ | 27 |
| Harvard Univ | Massachusetts Gen Hosp | 22 |
| Shanghai Jiao Tong Univ Rui Jin Hosp | Shanghai Jiao Tong Univ | 20 |
| Dankook Univ | Kangwon Natl Univ | 16 |
| Gangneung Wonju Natl Univ | Kangwon Natl Univ | 15 |
| Gangneung Wonju Natl Univ | Hallym Univ | 14 |
| Dankook Univ | Hallym Univ | 13 |
| Henry Ford Hlth Sci Ctr | Oakland Univ | 13 |
| Kangwon Natl Univ | Yonsei Univ | 13 |
| Air Force Mil Med Univ Xijing Hosp | Air Force Mil Med Univ | 11 |
| China Med Univ Hosp | China Med Univ | 11 |
| Nantong Univ Hosp | Nantong Univ | 11 |
| Seoul Natl Univ Hosp | Seoul Natl Univ | 11 |
| Toronto Western Hosp | Univ Toronto | 11 |
| Chinese Acad Med Sci | Peking Union Med Coll | 10 |

Supplementary Table 5 Institutions with more than 20 partners

| Rank | Institutions | Number of partners | Country |
| --- | --- | --- | --- |
| 1 | Massachusetts Gen Hosp | 33 | USA |
| 2 | Dzne | 32 | Germany |
| 3 | Univ Washington | 32 | USA |
| 4 | Johns Hopkins Univ | 29 | USA |
| 5 | Univ Calif Davis | 29 | USA |
| 6 | Univ Texas Houston | 29 | USA |
| 7 | Univ So Calif | 27 | USA |
| 8 | Mayo Clin | 26 | USA |
| 9 | Univ Bordeaux | 26 | France |
| 10 | Univ Miami | 26 | USA |
| 11 | Loma Linda Univ | 24 | USA |
| 12 | Univ Calif San Diego | 24 | USA |
| 13 | Univ Calif San Francisco | 24 | USA |
| 14 | Univ Edinburgh | 24 | Uk |
| 15 | Univ Calif Los Angeles | 23 | USA |
| 16 | Univ Gothenburg | 23 | Sweden |
| 17 | National Institute On Aging | 22 | USA |
| 18 | Univ Munich | 22 | Germany |
| 19 | Univ Oxford | 22 | UK |
| 20 | Karolinska Inst | 21 | Sweden |
| 21 | Max Planck Inst | 21 | Germany |
| 22 | Seoul Natl Univ | 21 | South Korea |
| 23 | Capital Med Univ Xuanwu Hosp | 20 | China |
| 24 | Univ Leipzig | 20 | Germany |
| 25 | Univ Michigan Ann Arbor | 20 | USA |
| 26 | Univ Queensland | 20 | Australia |
| 27 | Washington Univ | 20 | USA |

**Reference**

Amiry-Moghaddam, M., Otsuka, T., Hurn, P.D., Traystman, R.J., Haug, F.M., Froehner, S.C., et al. (2003) An alpha-syntrophin-dependent pool of AQP4 in astroglial end-feet confers bidirectional water flow between blood and brain. *Proc. Natl. Acad. Sci. U. S. A.* 100, 2106-2111. doi: 10.1073/pnas.0437946100

Badan, I., Buchhold, B., Hamm, A., Gratz, M., Walker, L.C., Platt, D., et al. (2003) Accelerated glial reactivity to stroke in aged rats correlates with reduced functional recovery. *J. Cereb. Blood Flow Metab.* 23, 845-854. doi: 10.1097/01.WCB.0000071883.63724. A7

Badaut, J., Hirt, L., Granziera, C., Bogousslavsky, J., Magistretti, P.J., and Regli, L. (2001). Astrocyte-specific expression of aquaporin-9 in mouse brain is increased after transient focal cerebral ischemia. *J. Cereb. Blood Flow Metab.* 21, 477-482. doi: 10.1097/00004647-20010500 0-00001

Bao, Y., Qin, L., Kim, E., Bhosle, S., Guo, H., Febbraio, M., et al. (2012). CD36 is involved in astrocyte activation and astroglial scar formation. *J. Cereb. Blood Flow Metab.* 32, 1567-1577. doi: 10.1038/jcbfm.2012.52

Barreto, G.E., Sun, X., Xu, L., and Giffard, R.G. (2011). Astrocyte proliferation following stroke in the mouse depends on distance from the infarct. *PLoS One* 6:e27881. doi: 10.1371/journal.pone.0027881

Chen, J., Sanberg, P.R., Li, Y., Wang, L., Lu, M., Willing, AE., et al. (2001). Intravenous administration of human umbilical cord blood reduces behavioral deficits after stroke in rats. *Stroke* 32, 2682-2688. doi: 10.1161/hs1101.098367

DePablo, Y., Nilsson, M., Pekna, M., and Pekny, M. (2013). Intermediate filaments are important for astrocyte response to oxidative stress induced by oxygen-glucose deprivation and reperfusion. *Histochem. Cell. Biol.* 140, 81-91. doi: 10.1007/s00418-013-1110-0

Ding, S., Wang, T., Cui, W., and Haydon, P.G. (2009). Photothrombosis ischemia stimulates a sustained astrocytic Ca2+ signaling in vivo. *Glia* 57, 767-776. doi: 10.1002/glia.20804

Garcia, J.H., Yoshida, Y., Chen, H., Li, Y., Zhang, Z.G., Lian, J., et al. (1993). Progression from ischemic injury to infarct following middle cerebral artery occlusion in the rat. *Am. J. Pathol.* 142, 623-635. doi: 10.1007/BF02899253

Hayakawa, K., Esposito, E., Wang, X., Terasaki, Y., Liu, Y., Xing, C., et al. (2016). Transfer of mitochondria from astrocytes to neurons after stroke. *Nature* 535, 551-555. doi: 10.1038/nature18928

Hayakawa, K., Nakano, T., Irie, K., Higuchi, S., Fujioka, M., Orito, K., et al. (2010). Inhibition of reactive astrocytes with fluorocitrate retards neurovascular remodeling and recovery after focal cerebral ischemia in mice. *J. Cereb. Blood Flow Metab.* 30, 871-882. doi: 10.1038/jcbfm.2009.257

Hayakawa, K., Pham, L.D., Katusic, Z.S., Arai, K., and Lo, E.H. (2012). Astrocytic high-mobility group box 1 promotes endothelial progenitor cell-mediated neurovascular remodeling during stroke recovery. *Proc. Natl. Acad. Sci. U. S. A.* 109, 7505-7510. doi: 10.1073/pnas.1121146109

Jeong, S.W., Chu, K., Jung, K.H., Kim, S.U., Kim, M., and Roh, J.K. (2003) Human neural stem cell transplantation promotes functional recovery in rats with experimental intracerebral hemorrhage. *Stroke* 34, 2258-2263. doi: 10.1161/01.STR.0000083698.20199.1F

Ji, C., Yu, X., Xu, W., Lenahan, C., Tu, S., and Shao, A. (2021). The role of glymphatic system in the cerebral edema formation after ischemic stroke. *Exp. Neurol.* 340:113685. doi: 10.1016/j.expneurol.2021.113685

Kawahara, K., Kosugi, T., Tanaka, M., Nakajima, T., and Yamada, T. (2005). Reversed operation of glutamate transporter GLT-1 is crucial to the development of preconditioning-induced ischemic tolerance of neurons in neuron/astrocyte co-cultures. *Glia* 49, 349-359. doi: 10.1002/glia.20114

Lyu, Z., Chan, Y., Li, Q., Zhang, Q., Liu, K., Xiang, J., et al. (2021) Destructive Effects of Pyroptosis on Homeostasis of Neuron Survival Associated with the Dysfunctional BBB-Glymphatic System and Amyloid-Beta Accumulation after Cerebral Ischemia/Reperfusion in Rats. *Neural. Plast.* 2021:4504363. doi: 10.1155/2021/4504363

Magnusson, J.P., Göritz, C., Tatarishvili, J., Dias, D.O., Smith, E.M., Lindvall, O., et al. (2014). A latent neurogenic program in astrocytes regulated by Notch signaling in the mouse. *Science* 346, 237-241. doi: 10.1126/science.346.6206.237

Manley, G.T., Fujimura, M., Ma, T., Noshita, N., Filiz, F., Bollen, A.W., et al. (2000). Aquaporin-4 deletion in mice reduces brain edema after acute water intoxication and ischemic stroke. *Nat. Med.* 6, 159-163. doi: 10.1038/72256

Matsui, T., Mori, T., Tateishi, N., Kagamiishi, Y., Satoh, S., Katsube, N., et al. (2002). Astrocytic activation and delayed infarct expansion after permanent focal ischemia in rats. Part I: enhanced astrocytic synthesis of s-100beta in the periinfarct area precedes delayed infarct expansion. *J. Cereb. Blood Flow Metab.* 22, 711-722. doi: 10.1097/00004647-200206000-00010

Michinaga, S., and Koyama, Y. (2019). Dual Roles of Astrocyte-Derived Factors in Regulation of Blood-Brain Barrier Function after Brain Damage. *Int. J. Mol. Sci.* 20:571. doi: 10.3390/ijms20030571

Nakase, T., Fushiki, S., and Naus, C.C. (2003). Astrocytic gap junctions composed of connexin 43 reduce apoptotic neuronal damage in cerebral ischemia. *Stroke* 34, 1987-1993. doi: 10.1161/01.STR.0000079814.72027.34

Overman, J.J., Clarkson, A.N., Wanner, I.B., Overman, W.T., Eckstein, I., Maguire, J.L., et al. (2012). A role for ephrin-A5 in axonal sprouting, recovery, and activity-dependent plasticity after stroke. *Proc. Natl. Acad. Sci. U. S. A.* 109(33):E2230-9. doi: 10.1073/pnas.1204386109

Pekny, M., and Nilsson, M. (2005). Astrocyte activation and reactive gliosis. *Glia* 50, 427-434. doi: 10.1002/glia.20207

Pekny, M., Pekna, M., Messing, A., Steinhäuser, C., Lee, J.M., Parpura, V., et al. (2016). Astrocytes: a central element in neurological diseases. *Acta Neuropathol.* 131, 323-345. doi: 10.1007/s00401-015-1513-1

Petito, C.K., and Babiak, T. (1982). Early proliferative changes in astrocytes in postischemic noninfarcted rat brain. *Ann. Neurol.* 11, 510–518. doi: 10.1002/ana.410110511

Petito, C.K., and Halaby, I.A. (1993). Relationship between ischemia and ischemic neuronal necrosis to astrocyte expression of glial fibrillary acidic protein. *Int. J. Dev. Neurosci.* 11, 239-247. doi: 10.1016/0736-5748(93)90082-o

Rakers, C., Schleif, M., Blank, N., Matušková, H., Ulas, T., Händler, K., et al. (2019) Stroke target identification guided by astrocyte transcriptome analysis. *Glia* 67, 619-633. doi: 10.1002/glia.23544

Risher, W.C., Andrew, R.D., and Kirov, S.A. (2009). Real-time passive volume responses of astrocytes to acute osmotic and ischemic stress in cortical slices and in vivo revealed by two-photon microscopy. *Glia* 57, 207-221. doi: 10.1002/glia.20747

Ruscher, K., Freyer, D., Karsch, M., Isaev, N., Megow, D., Sawitzki, B., et al. (2002). Erythropoietin is a paracrine mediator of ischemic tolerance in the brain: evidence from an in vitro model. *J. Neurosci.* 22, 10291-10301. doi: 10.1523/JNEUROSCI.22-23-10291.2002

Schroeter, M., Schiene, K., Kraemer, M., Hagemann, G., Weigel, H., Eysel, U.T., et al. (1995). Astroglial responses in photochemically induced focal ischemia of the rat cortex. *Exp. Brain Res.* 106, 1-6. doi: 10.1007/BF00241351

Shen, L.H., Li, Y., Gao, Q., Savant-Bhonsale, S., and Chopp, M. (2008). Down-regulation of neurocan expression in reactive astrocytes promotes axonal regeneration and facilitates the neurorestorative effects of bone marrow stromal cells in the ischemic rat brain. *Glia* 56, 1747-1754. doi: 10.1002/glia.20722

Shimada, I.S., Borders, A., Aronshtam, A., and Spees, J.L. (2011). Proliferating reactive astrocytes are regulated by Notch-1 in the peri-infarct area after stroke. *Stroke* 42, 3231-3237. doi:10.1161/STROKEAHA.111.623280

Sirko, S., Behrendt, G., Johansson, P.A., Tripathi, P., Costa, M., Bek, S., et al. (2013). Reactive glia in the injured brain acquire stem cell properties in response to sonic hedgehog. *Cell Stem Cell.* 12, 426-439. doi: 10.1016/j.stem.2013.01.019

Siushansian, R., Bechberger, J.F., Cechetto, D.F., Hachinski, V.C., and Naus, C.C. (2001). Connexin43 null mutation increases infarct size after stroke. *J. Comp. Neurol.* 440, 387-394. doi: 10.1002/cne.1392

Stoll, G., Jander, S., and Schroeter, M. (1998). Inflammation and glial responses in ischemic brain lesions. *Prog. Neurobiol.* 56, 149-171. doi: 10.1016/s0301-0082(98)00034-3

Yamashita, K., Vogel, P., Fritze, K., Back, T., Hossmann, K.A., and Wiessner, C. (1996). Monitoring the temporal and spatial activation pattern of astrocytes in focal cerebral ischemia using in situ hybridization to GFAP mRNA: comparison with sgp-2 and hsp70 mRNA and the effect of glutamate receptor antagonists. *Brain Res.* 735, 285-297. doi: 10.1016/0006-8993(96)00578-1

Zamanian, J.L., Xu, L., Foo, L.C., Nouri, N., Zhou, L., Giffard, R.G., and Barres, B.A. (2012). Genomic analysis of reactive astrogliosis. J*. Neurosci.* 32, 6391-6410. doi: 10.1523/JNEUROSCI.6221-11.2012

Zheng, W., Watts, L.T., Holstein, D.M., Wewer, J., and Lechleiter, J.D. (2013). P2Y1R-initiated, IP3R-dependent stimulation of astrocyte mitochondrial metabolism reduces and partially reverses ischemic neuronal damage in mouse. *J. Cereb. Blood Flow Metab.* 33, 600-611. doi: 10.1038/jcbfm.2012.214

Zhu, Z., Zhang, Q., Yu, Z., Zhang, L., Tian, D., Zhu, S., et al. (2007). Inhibiting cell cycle progression reduces reactive astrogliosis initiated by scratch injury in vitro and by cerebral ischemia in vivo. *Glia* 55, 546-558. doi: 10.1002/glia.20476
